# The Role of Physiotherapists in Multi-professional Primary Care: Lessons for Japan

**DOI:** 10.1298/ptr.R0043

**Published:** 2026-04-07

**Authors:** Stephen PECKHAM

**Affiliations:** Centre for Health Services Studies, University of Kent, United Kingdom

**Keywords:** Primary care, First-contact physiotherapy

## Abstract

Japan’s primary care system is evolving from a specialist-dominated model to one incorporating board-certified family physicians and generalist training pathways. However, the system remains fragmented, with limited integration of multi-professional teams. Primary care due to legal and systemic barriers, including the requirement for physician referrals and limited training for autonomous practice. Drawing on international evidence, this paper examines the potential role of physiotherapists as first-contact practitioners (FCPs) and advanced practice physiotherapists (APPs) within Japan’s evolving primary care system, with particular attention to system integration, workforce capacity, and care for musculoskeletal and chronic conditions. Studies demonstrate improved clinical outcomes, patient satisfaction, operational efficiency, and cost-effectiveness with physiotherapy-led models. FCPs reduce physician workload, medication use, and diagnostic imaging, while enhancing patient self-management and adherence to guidelines. However, challenges such as role ambiguity, diagnostic uncertainty, and inadequate funding hinder effective integration. Addressing these barriers requires legislative reform, comprehensive training, role clarity, and improved inter-professional collaboration. For Japan, embedding physiotherapists in multidisciplinary primary health care could support integrated community care, addressing the needs of an aging population and fragmented services. This transition necessitates redefining physiotherapy roles, expanding the scope of practice, and aligning with chronic care models. Coordinated policy initiatives on funding, training, and regulation are essential to develop multi-professional primary care teams and optimize patient outcomes. Lessons from international models provide valuable insights for advancing physiotherapy’s role in Japan’s primary care system.

## Introduction

Changing the skill mix of the primary care workforce has been proposed as a means of addressing the increasing public demand for primary care services, particularly in the context of an aging population and increasing numbers of people with long-term conditions. More generally, larger primary care multi-professional teams have been seen as a way of expanding the primary care workforce, especially where there are problems of primary care physician recruitment and retention^1)^. Skill mix is also seen as contributing to more responsive services, quality improvement, cost containment, and technological innovation^2,3)^. As such, given Japan’s developing primary care system, it is worth considering whether focusing primarily on small physician-led models or wider multi-professional models provides a potential pathway for meeting the increasing need for such services.

While increasing skill mix in primary care is a common feature internationally, the introduction of changes in skill mix in primary care settings is influenced by a range of contextual, political, social, and economic factors relevant to each individual health system. However, universally developing approaches to skill mix, alongside introducing changes in service delivery and organization, are considered appropriate and useful to support the provision of more accessible and responsive services—for example, increasing the number of appointments offered to patients and introducing new roles in primary care, as proposed in schemes in the United Kingdom (UK) and other countries^4)^. The range of additional roles is varied and can include extended nursing roles, pharmacists, community and social support workers, and physiotherapists. This paper specifically examines the inclusion of physiotherapists (usually called physical therapists in Japan) acting as first-contact practitioners (FCPs) or providing physiotherapy within primary care practice to patients referred to them within the practice group and what their role might be within the Japanese health care system as it develops a more universal and comprehensive primary care sector.

This paper argues that embedding physiotherapists within multidisciplinary primary care teams represents a feasible and potentially high-value strategy for addressing fragmentation, workforce pressures, and unmet rehabilitation needs in Japan. By examining international evidence on first-contact and advanced practice physiotherapy (APP), the paper assesses the implications for training, regulation, and funding within Japan’s developing primary care system. The first section provides an overview of Japanese primary care to set the wider context. This is followed by a summary of the international evidence and analysis of the contemporary practice of physiotherapy in primary care settings. The aim is to provide a broad assessment of key aspects of practice, such as clinical outcomes, patient experience, and operational aspects of provision. The paper then concludes with a section that examines key barriers and facilitators for developing physiotherapy in primary care and the relevance of this for developing the role of physiotherapy as part of future developments in Japanese primary care.

## Primary Care in Japan

Unlike Europe and North America, where primary care systems are well established with family physicians playing a central role, Japan’s health system has historically evolved without a formalized, gatekeeping primary care structure, which has implications for continuity of care, coordination across providers, and the organization of the primary care workforce^5)^. In an aging society, the increase in multimorbidity presents a challenge, as it can lead to fragmented care due to the specialization of medical services^6–8)^. Primary care in Japan is predominantly provided by physicians, but the system is only slowly evolving from a model where specialists handle most cases to one with a growing number of board-certified family physicians^9)^. Family medicine in Japan has been described as being an immature specialty, which is quite unusual for a developed country^10)^. It was only in 2010 that the Japan Primary Care Association (JPCA) was established to promote the practice of primary care. It provides continuous professional development and has become the recognized certifying body for primary care physicians, supporting the expansion of generalist training. More recently, reforms have focused on integrating generalist training pathways through the creation of a national specialty framework overseen by the Japanese Medical Specialty Board (JMSB), which has established certification criteria for the discipline of General Practice Specialist. The JPCA is now bringing its training and formal academic certifications to integrate with the *JMSB general practice pathway*.

Family physicians constitute less than 1% of all physicians, and primary care provision is fragmented, provided by a range of specialist practitioners, and delivered by mostly private, community-based clinics and small hospitals. In Japan, physicians are free to open their own practices regardless of their specialty or location^5,11)^. Funding for health care in Japan is primarily through its national health insurance scheme, and payments for primary care are based on a complex national fee-for-service schedule, which includes financial incentives for coordinating the care of patients with chronic diseases (known as Continuous Care Fees) and for team-based ambulatory and home care, but does not specifically designate family medicine separately. The schedule, set by the government, includes both primary and specialist services and has common prices for defined services, such as consultations, examinations, laboratory tests, imaging tests, and defined chronic disease management. Unlike many other countries, Japan’s health system does not designate clinics as “general practice clinics,” and there is not an official registry of general practice clinics tied to generalist certification—the designation is a medical specialism of the physician, not the clinic. In total, there are over 100000 clinics, of which some 83% are privately owned, with about 95% designated as providing “outpatient care” (i.e., no inpatient beds) and classified by medical specialty, such as obstetric care, internal medicine, pediatrics, and gastroenterology^9)^. Clinic staffing in Japan is highly skewed toward very small practices, with median staffing reflecting single-physician models in approximately 70%–75% of clinics, predominantly not registered as a primary care physicians, with the support of between 1 and 2 nurses^9,10)^.

Other professionals in primary care include nurses, pharmacists, dentists, and physiotherapists, with the JPCA leading the development of the specialty and its training programs, providing, for example, primary care nurse certification in 2019. Some larger older clinics employ dispensing pharmacists, and larger clinics may have other healthcare professionals to provide services such as radiography or social workers in mental health clinics^12)^. Currently, physiotherapists in Japan practicing within the national health insurance program provide physical therapy primarily within hospital settings (ca. 60% of all physiotherapists) or in other orthopedic, rehabilitation or pain clinics to provide rehabilitation, exercise therapy, and mobility training. This can be with family physician oversight, but less than 10% of physiotherapists work in primary care clinics^12)^.

Physiotherapists cannot provide physical therapy interventions to patients/users without the direction of an attending physician, and so in primary care settings, they currently cannot function as FCPs. Unlike many other countries, their practice scope is restricted by legislation, and training for autonomous practice is not provided. As a result, an appropriate role for physiotherapists in primary care has not been established despite the potential benefits and a growing need for physical therapy among patients^13)^. The reasons for this are complex and relate to the way physiotherapists are regulated in Japan and to the formal structure of primary care and first-contact services.

## The Role of the Physiotherapist in Primary Care

Internationally, there is a growing evidence base supporting the inclusion of physiotherapists in multidisciplinary primary care teams providing first-contact physiotherapy (FCP–also known as direct access physiotherapy [DAPT]) and APP. There is growing evidence of improved effectiveness across 4 areas of outcomes, such as clinical outcomes, patient experience, operational efficiency (systemic impact), and economic efficacy. Examples include improved management of patients with chronic diseases, increased patient satisfaction, and better outcomes for musculoskeletal (MSK) conditions regarding disabilitie^14–16)^. Other benefits include fewer consultations with their family physician, reduced healthcare costs, lower medication and imaging use, and better quality of life than for patients seeing a physician first^16–21)^. APP models also demonstrate positive results in efficiency, cost, and patient satisfaction.

First-contact physiotherapists are expert physiotherapists who undertake the first patient consultation in a primary care setting; while predominantly for MSK, their role includes rehabilitation and support for people with chronic health conditions. FCP is an emerging model of care where the specialist physiotherapist, located within or associated with general practice, performs the initial patient assessment, diagnosis, and management^22)^. The fundamental characteristic of the FCP role is the ability to provide direct access to a physiotherapist without the need for a prior assessment or referral from a physician^1,22)^.

APPs are those practitioners who have developed their skills and knowledge in an area of practice through clinical experience and postgraduate study and demonstrated achievement of specific competencies^23)^. APPs apply in-depth, research-informed knowledge and skills to physiotherapy assessment and care, understanding the person, their context and the influences on their health to inform diagnosis, management, and prognosis. Advanced practitioners may work in primary or secondary care settings. Physiotherapists and other healthcare professionals can refer clients to APPs when they need a clinical case review or advanced assessment and management. A key role for APPs is to triage cases referred to specialist clinics to ensure active rehabilitation options are appropriately explored prior to, or in conjunction with, consideration of surgical or other more specialist approaches.

Internationally, there is a growing evidence base that supports the role of physiotherapists as primary assessors for common aches and pains, offering a practical and financially expedient solution to mounting pressures within Japanese primary care. The following discussion draws on this evidence to highlight potential areas of benefit that support the inclusion of physiotherapists in developing primary care in Japan.

### Clinical outcomes

Overall, moderate-quality evidence indicates that first-contact physiotherapy achieves clinical outcomes comparable to, and in some domains superior to, physician-led care for MSK conditions, particularly in functional status and health-related quality of life^14,24,25)^. Evidence reviews consistently suggest that FCP can provide better outcomes in terms of disability and quality of life for patients with MSK disease compared to primary physician-led usual medical care^14,20,24)^. While some studies have found no significant differences in pain reduction between FCP and usual medical care, the evidence does suggest better clinical outcomes in FCP patients related to function and health-related quality of life. The magnitude of the differences observed in disability and quality of life have been shown to be clinically important. In their evaluation of the FCP model, Stynes et al. showed that 54% of patients achieved a minimal important change (6 points or more) on the MSK Health Questionnaire at 3 months follow-up, meeting a predefined success criterion^26)^. However, another study found similar clinical improvements in disability and pain over 3 months between the patients with acute low back pain (LBP) cared for by family practitioners and those cared for by a physiotherapist^27)^. Crucially though, the study found that physiotherapists prescribed significantly fewer medications than family physicians, suggesting the FCP model may offer a path to reduce unnecessary resource use while maintaining high-quality patient care.

Earlier data from a national evaluation in England highlighted issues about the comparability of patient characteristics between physiotherapist and family physician care, with the latter appearing to handle more complex cases^26)^. However, in their later study of physiotherapy in 46 primary care practices in England, Walsh and colleagues found that FCP-led models of care for patients with MSK provided safe, clinically effective patient management and reduced opioid use in the cohort of patients surveyed, echoing the findings of studies in other countries^27,28)^. While there is clear evidence of good outcomes when compared to family practitioner care, some studies suggest that some physiotherapists experience diagnostic challenges, raising concerns about missing serious pathology; Budtz and colleagues found some physiotherapists lacked the ability to make correct management decisions in critical medical categories^29,30)^.

### Patient experience

In the UK, Goodwin and Hendrick found that family physicians expressed high confidence in the FCPs’ competence and patients reported a positive experience characterized by improved self-management and specialized advice^31)^. This reflected the findings of an earlier 2012 Swedish study by Ludvigsson and Enthoven, which found that 80% of the patients who saw the physiotherapist reported complete satisfaction with the information they received and their confidence in the physiotherapists’ competency to assess their problem^32)^. The evidence from reviews of physiotherapist-led care provides strong confirmation of high levels of patient satisfaction and effective management of chronic conditions, particularly for LBP and significantly higher compared to primary care physician-led care^20,33)^. Studies have found that physiotherapists bring particular expertise in the management of chronic diseases and MSK conditions within primary care settings^34)^. Patients report that physiotherapist-led primary care models for LBP have helped improve their confidence in managing LBP and ensure guideline-adherent care^34)^. Patients appreciated that the physiotherapist can develop a strong therapeutic alliance by being genuine, present, and providing comprehensive, thorough, and tailored care^34)^. They also found that co-location of the physiotherapist within the primary care site was valued because it offered improved access and convenience, leading to improved patient engagement.

### System efficiency and costs

There is consistent evidence from studies in Europe, North America, and Australasia that FCP and APP models improve system efficiency by reducing demand on physician time, lowering overall episode costs, and decreasing reliance on pharmacological and diagnostic interventions for MSK conditions. By enabling physiotherapists to function as primary assessors and managers of care, these models allow a substantial proportion of patients to be managed entirely within primary care, both relieving pressure on family physicians and reducing pressure on specialist services^20,27,35)^.

A 4-year evaluation of FCP implementation within a UK primary care network demonstrated that 86.4% of patients were managed without onward referral beyond primary care, indicating a substantial reduction in family physician workload^35)^. Referral rates to orthopedics by FCPs were comparable to, and in some cases lower than, those reported for family physicians, suggesting that physiotherapists were able to appropriately manage complexity and escalate care when required^27,35)^. However, national evaluations in the UK have also shown that the full potential of FCP to reduce physician workload may be constrained by limited service capacity, variable funding arrangements, and inconsistent patient awareness of direct access pathways^22,36)^. These findings highlight the importance of adequate workforce planning and system-level support in realizing efficiency gains.

Comparable results have been observed internationally. In a pragmatic cluster randomized controlled trial conducted in French primary care, physiotherapists acting as FCPs for patients with acute LBP prescribed significantly less medication than family physicians while achieving comparable clinical outcomes^28)^. Family physicians involved in the task-sharing model reported high confidence in physiotherapists’ diagnostic and management capabilities, and the model was perceived as a promising strategy for addressing workforce shortages in MSK care^37)^. Systematic reviews similarly report that patients managed through DAPT are less likely to require follow-up consultations with physicians and experience fewer referrals for specialist care^20,33)^.

Economic evaluations further support the efficiency of FCP and APP models. Studies consistently demonstrate lower average costs per episode of care for patients managed by physiotherapists compared with physician-led usual care, even when physiotherapy consultations are longer in duration^20,27,33)^. Cost savings are largely attributable to reduced prescribing—particularly of analgesics and opioids—lower utilization of diagnostic imaging, and fewer referrals to orthopedic and secondary care services^20,27)^. For example, Walsh et al. reported that overall NHS costs over a 6-month follow-up period were approximately two and a half times higher in physician-led care compared with FCP and APP models, driven primarily by downstream resource use^27)^.

Importantly, these efficiency and cost benefits do not appear to be achieved at the expense of care quality or safety. Rates of re-consultation for the same condition are consistently lower among patients initially managed by FCPs compared with those seen by family physicians, and onward referral patterns suggest appropriate clinical decision-making rather than under-referral.^27,35)^. Moreover, physiotherapy-led models are associated with greater adherence to evidence-based guidelines, particularly those promoting active management and self-management approaches for MSK disorders, which may contribute to both improved outcomes and reduced unnecessary healthcare utilization^20,33,34)^.

Overall, the evidence indicates that FCP and APP models offer a cost-effective and system-efficient approach to managing MSK and selected chronic conditions within primary care. For health systems facing workforce shortages and increasing demand, these models provide a means of improving productivity while maintaining high standards of patient care—an issue of particular relevance for the future development of primary care in Japan.

### Implementation barriers and challenges

Despite strong international evidence supporting the integration of physiotherapists into primary care, the evidence also highlights that implementation has been constrained by 3 interrelated structural barriers: restricted scope of practice, lack of role clarity within multidisciplinary teams, and misalignment between funding mechanisms and service delivery^21,24,29)^. These constraints shape secondary challenges frequently reported in the literature, including high workload pressures, diagnostic uncertainty, and weak interprofessional collaboration, which are better understood as consequences of system design rather than limitations of individual practitioners^14,23,24)^. Having a clear understanding of these challenges will be particularly important for policymakers in considering how to take forward primary care development in Japan. Importantly, it also highlights the need to address not just structures of service delivery but also the institutional and regulatory frameworks for education and training, funding, and clinical practice.

Addressing these barriers requires the development of comprehensive education and training programs that extend beyond profession-specific competencies. Training should strengthen physiotherapists’ capabilities in evidence-based practice, clinical reasoning, and decision-making under uncertainty, while also supporting collaborative, team-based models of care^24,38)^. Education must be reoriented toward empowering patients, promoting self-management, and embedding person-centered consultation approaches within routine practice^24)^. Strategies shown to mitigate diagnostic uncertainty include the use of safety-netting, watchful waiting, and structured escalation pathways, alongside opportunities for reflective practice and clinical supervision^24,29)^. Access to both formal support mechanisms—such as mentorship, debriefing, and management support—and informal peer networks has been shown to reduce professional isolation and improve clinician confidence^24,29)^. This has important implications for the development of practice in Japan, given the current fragmentation of services and reliance on small private practices. Evidence from the introduction of APP roles in New Zealand further highlights the importance of nonacademic attributes, including professionalism, empathy, and communication skills, alongside advanced clinical training, suggesting that reforms in both physiotherapy and medical education are likely to be required in Japan^23)^.

Inadequate funding arrangements and poorly aligned payment mechanisms represent a further major barrier to implementation^24)^. Evidence from Australian private practice highlights dissatisfaction with the limitations of the Medicare Chronic Disease Management program in supporting sustained interprofessional collaborative practice^23)^. More broadly, successful integration of physiotherapists into primary care depends on funding models that explicitly recognize multidisciplinary service delivery, provide protected time for collaboration, and incentivize appropriate referral and case management^23,24)^. For Japan, this may necessitate revisions to the national health insurance scheme to support defined rehabilitation service packages, introduce targeted funding streams, or adopt payment mechanisms that reward coordination and value-based care.

Clarifying professional roles and responsibilities across the primary care team is repeatedly identified as a critical enabler of successful integration^24)^. Clear delineation between generalist, advanced practice, and specialist physiotherapy roles is essential to avoid role confusion and underutilization of skills^14)^. This should be supported by explicit referral pathways, agreed scopes of practice, and shared clinical governance arrangements. Professional advocacy and strong organizational leadership are also necessary to raise awareness among policymakers and secure political support for expanded roles, with greater participation of physiotherapists in policy development processes identified as a key facilitator^24)^. Strengthening intra- and interprofessional collaboration requires deliberate investment in communication structures, shared learning opportunities, and team-based working practices within primary care, which are still to be developed in Japan^24)^.

Additional facilitators of integration relate to technology, workflow design, and clinical approach. Secure, interoperable digital communication systems are essential to support effective interprofessional collaborative practice and ensure timely information exchange across providers^24,39)^. Supporting infrastructure—such as national quality registers, surveillance systems, and integrated electronic health records—can further enhance coordination and accountability^24)^. It will also require significant changes to the organization and structure of Japanese primary care services, which currently operate as independent clinics. Locating FCP services within or alongside family physician practices has been shown to improve communication, trust, and engagement among professionals and patients^22,28,37)^. Finally, physiotherapists must adopt a biopsychosocial approach to care, supported by appropriate physical space, workflow design, and staff rotation strategies that facilitate familiarity with diverse clinical environments and patient populations^23)^.

## Developing Physiotherapy in Japanese Primary Care

Lessons from the development of primary care-based physiotherapy in other countries will therefore require both a reassessment of how primary care will develop and the role of physiotherapists. The future development of primary care in Japan requires physiotherapists to fundamentally redefine their role, shifting from a primarily hospital-based, specialized rehabilitation model to one that is integrated, community-focused, and autonomous^13)^. This expansion is necessary to address the country’s aging population, fragmented medical services, and the narrow scope of practice often seen among primary care physicians^9)^. The environment and expectations for physiotherapists in Japan are significantly different from other developed healthcare systems from which the majority of evidence supporting their inclusion in primary care teams has been drawn. This distinct difference is due to 3 broad historical and structural factors: systemic restriction, views about the role of physiotherapists, and underdeveloped primary care infrastructure.

Currently, the involvement of Japanese physiotherapists in public health is limited by legal restrictions and primarily confined to individual rehabilitation under a physician’s direction. For physiotherapists to significantly develop within primary care, this legal and professional scope must be expanded to serve broader community health goals^40)^. In Japan, physiotherapists can only accept patients who are referred to them by registered doctors, and as a result, training for autonomous practice is generally not provided. The requirement in Japanese legislation for patients to obtain referrals from registered doctors before consulting physiotherapists presents a significant barrier to first-point-of-contact care by physiotherapists. As legislation also defines the scope of practice of physiotherapists, this also places restrictions on advanced practice. The medical system is also highly fragmented, lacking a centralized patient registration scheme, which impedes practice continuity.

This statutory structure has influenced why primary care physicians view physiotherapists as specialists focused on physical structure and rehabilitation within a mono-causal viewpoint^13)^. Physicians viewed physiotherapists as specialists in physical structural aspects (like muscle strength and joint range of motion) and action capabilities (like walking). The physiotherapist’s role is defined as achieving a specific and well-defined outcome for the individual, such as increasing the walking function of inpatients to help them return home on foot^13)^. This traditional role has developed through the adaptation of physiotherapy practice to the hospital environment, often defined by a short clinical course for improvement or maintenance, coinciding with the limited hospitalization period specified under Japan’s flat-fee payment system for acute inpatient care. This hospital-oriented view of physiotherapy reflects the dominance of such provision. In 2019, physiotherapy employment in Japan remained institutionally concentrated, with the majority of practitioners based in hospital settings (59%), followed by homes (18%), nursing homes (9%), and clinics (8%)^13)^. Consistent with the historical organization of the Japanese health system, hospitals continue to play a substantial role in outpatient care, delivering both primary- and secondary-level services. National estimates suggest that roughly one-third of outpatient encounters occur in hospital settings rather than clinics^5)^.

However, this “traditional” view about physiotherapy practice did not seem to limit the views of primary care physicians about the future potential role of physiotherapists in primary care settings^13)^. There was a clear expectation that physiotherapists in primary care should adopt a broader, continuous role aligned with integrated community care (“Rehabilitation in integrated community care”). Given the growing number of persons with complex and long-term health needs, Japanese primary health care will need, like many health care jurisdictions, to consider systems of care guided by chronic care models, in which self-management is a key component of optimizing health status and service delivery^41–43)^. Allen et al. argue that developing primary care models

“… that use health workers trained in family medicine and include multidisciplinary teams are the most likely to be delivering the core functions of primary care and are best positioned to face the challenges of the future compared with models based on independent practitioners with limited family medicine training.” (p. 4)^44)^

However, attention also needs to be paid to avoiding fragmentation, which can disrupt the holistic management and continuity of care^44)^. As part of system reform, developing primary care and creating a clearer distinction between primary care and hospital outpatients (including revision of the payment system) has been suggested as a way to address the changing epidemiological and demographic context of health care in Japan^45)^. Embedding physiotherapists in publicly funded primary health care settings might be a cost-effective and value-based strategy for providing more tailored, comprehensive, holistic care, but will require revision of the national health insurance scheme.

This strategy would enable physiotherapists to provide comprehensive care and self-management support that is focused on promoting health and preventing disease, screening and early detection, assessing and managing chronic disease, and navigating the health system and managing their patient caseload^14,24)^. The evidence from research in other countries suggests that physiotherapists engage in collaborative practice with a diverse range of health professionals, including occupational therapists, physical trainers, nutritionists, orthotists, social workers, nurses, and, of course, physicians^14)^. However, studies have also identified integration barriers for physiotherapists, primarily due to a misunderstanding of their roles by other primary care team members highlighting the need to invest in training and education within a multi-professional context^24,46)^. The collaboration between physiotherapists and physicians can be beneficial in optimizing the care provided to patients with specific conditions, such as MSK, and LBP in particular, and long-term conditions such as chronic obstructive pulmonary disease^47)^. Similarly, collaboration within a larger primary care team, comprising nurses, physicians, and occupational therapists, ensures that a more comprehensive rehabilitation approach provides benefits to patients^46)^.

## Conclusions

Primary care-based first-contact physiotherapy models consistently demonstrate clinical noninferiority compared to traditional physician-led care for MSK disorders, providing equivalent or superior outcomes in terms of physical function and quality of life. They can result in a more efficient use of healthcare resources by significantly reducing the proportion of medication prescriptions—including opioid derivatives—and decreasing the reliance on expensive diagnostic imaging and specialist referrals. From an economic perspective, FCP models are cost-effective when compared to family physician-led care. Patients managed under these systems report overwhelmingly positive experiences, characterized by high satisfaction rates, significantly shorter waiting times, and an enhanced sense of self-efficacy in managing their own health.

However, the successful implementation of these roles requires addressing persistent barriers to integration, such as the lack of professional role clarity, insufficient knowledge among family physicians regarding the full scope of physiotherapy expertise, and the diagnostic uncertainty felt by clinicians managing undifferentiated presentations. A cultural shift is necessary to move away from fragmented “isolated silos” toward integrated community-based care that supports interprofessional collaborative practice. Standardized career pathways and advanced certifications, such as those established in New Zealand and the UK, are vital for clinician retention and ensuring stakeholders have confidence in the advanced clinical competencies of the workforce. Finally, in countries with less mature primary care systems like Japan, overcoming the traditional hospital-centric perception of the physiotherapist as a technician focused solely on well-defined functional outcomes is essential for transitioning to a more holistic, integrated care model.

## References

[1] Zouch JH, Berg B, et al.: Reducing strain on primary healthcare systems through innovative models of care: the impact of direct access physiotherapy for musculoskeletal conditions—an interrupted time series analysis. Fam Med Community Health. 2024; 12: e002998. 10.1136/fmch-2024-002998 PMC11423733 39317459

[2] Sibbald B, Shen J, et al.: Changing the skill-mix of the health care workforce. J Health Serv Res Policy. 2004; 9(Suppl. 1):28–38. 10.1258/135581904322724112 15006226

[3] Francetic I, Gibson J, et al.: Skill-mix change and outcomes in primary care: longitudinal analysis of general practices in England 2015-2019. Soc Sci Med. 2022; 308: 115224. 10.1016/j.socscimed.2022.115224 35872540

[4] Maier CB, Kroezen M, et al. (eds): Skill-mix innovation, effectiveness and implementation: improving primary and chronic care. Cambridge: Cambridge University Press; 2022.

[5] Kato D, Ryu H, et al.: Building primary care in Japan: literature review. J Gen Fam Med. 2019; 20: 170–179. 10.1002/jgf2.252 PMC6732569 31516802

[6] Ando T, Sasaki T, et al.: Measurement of polydoctoring as a crucial component of fragmentation of care among patients with multimorbidity: cross-sectional study in Japan. J Gen Fam Med. 2023; 24: 343–349. 10.1002/jgf2.651 PMC10646296 38025930

[7] Kuzuya M: Era of geriatric medical challenges: multimorbidity among older patients. Geriatr Gerontol Int. 2019; 19: 699–704. 10.1111/ggi.13742 31397060

[8] Akintayo-Usman NO: Fragmentation of care: a major challenge for older people living with multimorbidity. Geriatr Gerontol Aging. 2021; 15: e0210030.

[9] Higuchi T, Nakamura M, et al.: Scope of practice of Japanese primary care physicians and its associated factors: a cross-sectional study. Fam Med Community Health. 2025; 13: e003191. 10.1136/fmch-2024-003191 PMC11795410 39900377

[10] Okazaki Y, Yoshida S, et al.: Geographic distribution of family physicians in Japan and the USA: a cross-sectional comparative study. Rural Remote Health. 2022; 22: 7163. 10.22605/RRH7163 35706356

[11] World Health Organization: *Human resources for health country profiles: Japan*. Geneva: WHO; 2017.

[12] Haruta J, Goto R: Exploring factors associated with healthcare professionals’ subjective perceptions of complex issues in primary care in Japan: a self-administered survey study on confidence, satisfaction and burden levels. BMJ Open. 2024; 14: e081328. 10.1136/bmjopen-2023-081328 PMC10966809 38531578

[13] Goto R, Haruta J, et al.: What role expectations do primary care physicians in Japan hold for physical therapists regarding primary care? J Prim Care Community Health. 2022; 13: 21501319221124316. 10.1177/21501319221124316 PMC9647272 36345202

[14] Champoux M, Poirier A, et al.: Roles of physiotherapists in primary care teams: a scoping review. BMJ Open. 2025; 15: e092276. 10.1136/bmjopen-2024-092276 PMC11808882 39920051

[15] McColl MA, Shortt S, et al.: Models for integrating rehabilitation and primary care: a scoping study. Arch Phys Med Rehabil. 2009; 90: 1523–1531. 10.1016/j.apmr.2009.03.017 19735780

[16] Pinnington MA, Miller J, et al.: An evaluation of prompt access to physiotherapy in the management of low back pain in primary care. Fam Pract. 2004; 21: 372–380. 10.1093/fampra/cmh406 15249525

[17] Holdsworth LK, Webster VS, et al.: What are the costs to NHS Scotland of self-referral to physiotherapy? Results of a national trial. Physiother. 2007; 93: 3–11.

[18] Ojha HA, Snyder RS, et al.: Direct access compared with referred physical therapy episodes of care: a systematic review. Phys Ther. 2014; 94: 14–30. 10.2522/ptj.20130096 24029295

[19] Bornhöft L, Larsson MEH, et al.: Physiotherapy in Primary Care Triage – the effects on utilization of medical services at primary health care clinics by patients and sub-groups of patients with musculoskeletal disorders: a case-control study. Physiother Theory Pract. 2015; 31: 45–52. 10.3109/09593985.2014.932035 24988315

[20] Demont A, Bourmaud A, et al.: The impact of direct access physiotherapy compared to primary care physician led usual care for patients with musculoskeletal disorders: a systematic review of the literature. Disabil Rehabil. 2021; 43: 1637–1648. 10.1080/09638288.2019.1674388 31603709

[21] Bodenheimer T, Kucksdorf J, et al.: Integrating physical therapists into primary care within a large health care system. J Am Board Fam Med. 2021; 34: 866–870. 10.3122/jabfm.2021.04.200432 34312283

[22] Goodwin R, Moffatt F, et al.: Evaluation of the First Contact Physiotherapy (FCP) model of primary care: a qualitative insight. Physiotherapy. 2021; 113: 209–216. 10.1016/j.physio.2021.08.003 PMC8612276 34583834

[23] Stotter G, McKinlay E, et al.: Advanced practice physiotherapists in primary health care: stakeholders’ views of a new scope of practice. J Prim Health Care. 2024; 16: 160–169. 10.1071/HC24029 38941257

[24] ShahAli S, Shahabi S, et al.: Barriers and facilitators of integrating physiotherapy into primary health care settings: a systematic scoping review of qualitative research. Heliyon. 2023; 9: e20736. 10.1016/j.heliyon.2023.e20736 PMC10582494 37860510

[25] Oshomoji OI, Ajiroba JO. The effect of community-based physiotherapy programs on chronic disease management in rural settings: a systematic review. J Adv Med Biomed Res. 2025; 33: 179–191.

[26] Stynes S, Jordan KP, et al.: Evaluation of the First Contact Physiotherapy (FCP) model of primary care: patient characteristics and outcomes. Physiotherapy. 2021; 113: 199–208. 10.1016/j.physio.2021.08.002 34656297

[27] Walsh NE, Halls S, et al.: First contact physiotherapy: an evaluation of clinical effectiveness and costs. Br J Gen Pract. 2024; 74: e717–e726. 10.3399/BJGP.2023.0560 PMC11325442 38429110

[28] Kechichian A, Desmeules F, et al.: Physiotherapists as first-contact practitioners for patients with low back pain in French primary care: a pragmatic cluster randomised controlled trial. BMC Health Serv Res. 2024; 24: 1427. 10.1186/s12913-024-11814-2 PMC11572111 39558330

[29] Ingram S, Stenner R, et al.: The experiences of uncertainty amongst musculoskeletal physiotherapists in first contact practitioner roles within primary care. Musculoskeletal Care. 2023; 21: 644–654. 10.1002/msc.1735 36683250

[30] Budtz CR, Hansen RP, et al.: The prevalence of serious pathology in musculoskeletal physiotherapy patients – a nationwide register-based cohort study. Physiother. 2021; 112: 96–102. 10.1016/j.physio.2021.03.004 34034209

[31] Goodwin RW, Hendrick PA. Physiotherapy as a first point of contact in general practice: a solution to a growing problem? Prim Health Care Res Dev. 2016; 17: 489–502. 10.1017/S1463423616000189 27263326

[32] Ludvigsson ML, Enthoven P. Evaluation of physiotherapists as primary assessors of patients with musculoskeletal disorders seeking primary health care. Physiother. 2012; 98: 131–137. 10.1016/j.physio.2011.04.354 22507363

[33] Gallotti M, Campagnola B, et al.: Effectiveness and consequences of direct access in physiotherapy: a systematic review. J Clin Med. 2023; 12: 5832. 10.3390/jcm12185832 PMC10531538 37762773

[34] Vader K, Donnelly C, et al.: Implementing a new physiotherapist-led primary care model for low back pain: a qualitative study of patient and primary care team perspectives. BMC Prim Care. 2022; 23: 201. 10.1186/s12875-022-01817-5 PMC9367061 35948876

[35] Henning M, Henning R, et al.: First contact physiotherapy: a 4-year service evaluation of UK primary care data. Musculoskeletal Care. 2024; 22: e1961. 10.1002/msc.1961 39394651

[36] Halls S, Thomas R, et al.: Provision of first contact physiotherapy in primary care across the UK: a survey of the service. Physiotherapy. 2020; 108: 2–9. 10.1016/j.physio.2020.04.005 32693238

[37] Kechichian A, Desmeules F, et al.: Acceptability of a task sharing and shifting model between family physicians and physiotherapists in French multidisciplinary primary healthcare centres: a cross-sectional survey. Fam Med Community Health. 2022; 10: e001644. 10.1136/fmch-2022-001644 PMC9204406 35710147

[38] Seaton J, Jones A, et al.: Physiotherapy private practitioners’ opinions regarding interprofessional collaborative practice: a qualitative study. J Interprof Care. 2024; 38: 10–21. 10.1080/13561820.2023.2221687 37288950

[39] Seaton J, Jones A, et al.: Promoting effective interprofessional collaborative practice in the primary care setting: recommendations from Queensland physiotherapy private practitioners. Aust J Prim Health. 2024; 30: PY23175. 10.1071/PY23175 38310645

[40] Kimura A. Issues in physiotherapy in Japanese public health from the perspective of differences with other countries. Jpn J Public Health Phys Ther. 2024; 11: 6–14.

[41] Barr VJ, Robinson S, et al.: The expanded Chronic Care Model: an integration of concepts and strategies from population health promotion and the Chronic Care Model. Hosp Q. 2003; 7: 73–82. 10.12927/hcq.2003.16763 14674182

[42] Kennedy A, Rogers A, et al.: Support for self care for patients with chronic disease. BMJ. 2007; 335: 968–970. 10.1136/bmj.39372.540903.94 PMC2071971 17991978

[43] Morgan MW, Zamora NE, et al.: An inconvenient truth: a sustainable healthcare system requires chronic disease prevention and management transformation. Healthc Pap. 2007; 7: 6–23. 10.12927/hcpap.2007.18992 17595546

[44] Allen LN, Rasanathan K, et al.: Models of global primary care post-2030. Lancet Prim Care. 2025; 1: 100027. 10.1016/j.lanprc.2025.100027 PMC12485077 41040968

[45] Le KD: Promoting health system resilience through health policy reform for the ageing population of Japan: a rapid literature review. Cureus. 2025; 17: e90931. 10.7759/cureus.90931 PMC12458914 41001327

[46] Törnudd ML, Peolsson A, et al.: The perceptions of nurses and physicians in primary care of rehabilitation for frail older adults. Clin Rehabil. 2024; 38: 1276–1286. 10.1177/02692155241258286 PMC11468118 38825588

[47] Pagano L, McKeough Z, et al.: Acceptability and barriers of a GP-physiotherapist partnership in the diagnosis and management of COPD in primary care: a qualitative study. Health Expect. 2024; 27: e13935. 10.1111/hex.13935 PMC10757211 38063819

